# Impact of a sports nutrition education intervention on knowledge and dietary behavior in NCAA Division I athletes

**DOI:** 10.1080/15502783.2025.2550164

**Published:** 2025-08-30

**Authors:** Mason Margut, Celia Lemieux, Sophie Gulleyan, Ethan Werlau, Kimberly Michelle Singleton, Jamie McAllister-Deitrick

**Affiliations:** Coastal Carolina University, Department of Kinesiology, Conway, SC, USA

**Keywords:** Dietary behavior, athlete, sport nutrition, intervention

## Abstract

**Background:**

Nutrition is integral in performance and recovery of athletes. Research has found collegiate athletes have a lack of nutritional understanding and their diets are typically insufficient. Due to this lack of understanding and improper nutrition intake, implementation of a sports nutrition education intervention (SNEI) may benefit an athlete’s performance. The purpose of this study was to investigate the effects of an SNEI on sports nutrition knowledge (SNK) and dietary behavior in Division I (DI) athletes.

**Methods:**

Fourteen NCAA DI male soccer players participated during 10-weeks of in-season training/matches. Athletes received the SNEI at baseline, as well as biweekly reminders throughout the season. Athletes completed a validated questionnaire assessing SNK and self-perceived questionnaires assessing dietary behavior and recovery pre- and post-intervention.

**Results:**

Paired samples t-tests revealed significant improvement in sports nutrition knowledge post SNEI (t(13) = −3.52, *p* < .01). There were no significant differences in dietary behavior post SNEI (t(13) = 1.01, *p* = .331).

**Conclusions:**

The lack of significant changes in dietary behavior could be influenced by a myriad of factors best explained through a theoretical approach, such as the Theory of Planned Behavior, which suggests that perceived control may impact an athlete’s dietary choices. Similarly, using Social Cognitive Theory may highlight a potential lack of self-efficacy required to make proper nutritional choices. The findings generally support the use of SNEI to increase SNK; however, results indicate a need for future interventions to further investigate actual dietary intake. Further research exploring athletes’ perceived control and self-efficacy may help better explain dietary behavior beyond their understanding of sport nutrition.

